# Baicalin Attenuates Brain Edema in a Rat Model of Intracerebral Hemorrhage

**DOI:** 10.1007/s10753-013-9717-9

**Published:** 2013-08-25

**Authors:** Qing-Bo Zhou, Yun-Ling Jin, Qing Jia, Yuan Zhang, Lu-Yang Li, Ping Liu, Yuan-Tao Liu

**Affiliations:** 1 Department of Neurology, The Second Hospital, Shandong University, Jinan, 250033 China; 2 Department of Endocrinology, The Second Hospital, Shandong University, 247 Beiyuan Road, Jinan, Shandong 250033 China; 3 Department of Experimental Pathology, Institute of Basic Medicine, Shandong Academy of Medical Sciences, Jinan, 250012 China; 4 Department of Neurology, Qilu Hospital, Shandong University, Jinan, 250012 China; 5 Department of Pharmacology, College of Medicine, Shandong University, Jinan, 250012 China

**Keywords:** baicalin, intracerebral hemorrhage, brain edema, nuclear factor-κB, metalloproteinase-9

## Abstract

Baicalin is a flavonoid compound purified from the roots of *Scutellaria baicalensis*, which possesses multiple biological activities. Previous studies have shown that baicalin is protective in ischemic cerebral diseases. The aim of the present study was to examine the effects of baicalin on brain injury in a rat model of intracerebral hemorrhage (ICH) and to explore the possible mechanisms. Intracerebral hemorrhage was induced in male Wistar rats by injection of 0.5 U collagenaseVII to the caudate nucleus. Sham operation rats were injected with equal volume of saline. After the induction of ICH, the rats were randomly divided into four groups and administered with different dose of baicalin (0, 25, 50, or 100 mg/kg in saline) through peritoneal injection. The brain tissues around the hemorrhage areas were collected on days 1, 3, and 5 after treatment. Brain edema was analyzed by desiccation method; the metalloproteinase-9 (MMP-9) protein and mRNA expression were determined by western blotting and real time RT-PCR, respectively. Nuclear factor-κB (NF-κB) protein expression was analyzed by western blotting. IL-1β and IL-6 levels were determined by enzyme-linked immunosorbent assay. Blood–brain barrier permeability was determined by Evans blue leakage method. The results showed that baicalin reduced brain edema following ICH in a dose-dependent manner, with concomitant inhibition of NF-κB activation and suppression of MMP-9 expression. In addition, baicalin also reduced IL-1β and IL-6 production, as well as blood–brain barrier permeability. The above results indicated that baicalin prevents against perihematomal edema development after intracerebral hemorrhage possibly through an anti-inflammatory mechanism.

## INTRODUCTION

Intracerebral hemorrhage (ICH) is one of the most devastating types of stroke, accounting for approximately 10–15 % of all strokes. Patients with ICH have poor prognosis, 80 % of those survivors beyond the acute phase may suffer prolonged neurological deficits and brain atrophies [1]. So far, the management of ICH is generally supportive as there are no specific treatments shown to improve the outcome of ICH [2].

Brain damage after ICH is caused not only by the mass effect of hematoma but also by the secondary pathological processes. ICH causes secondary injury through various pathways, including the initiation of an acute inflammatory response, local release of reactive oxygen species (ROS), breakdown of blood–brain barrier (BBB), and perihematomal edema [3, 4]. Matrix metalloproteinases (MMPs) are a family of proteolytic enzymes that increase BBB permeability by cleavage of microvascular extracellular matrix and protein components. Previous studies have implicated MMPs in BBB disruption after cerebral ischemia [5, 6]. So far, accumulating evidence has shown that MMPs, in particular MMP-9, may play an important role in ICH-induced secondary brain injury [7]. Clinical studies have shown that the blood MMP-9 level is elevated in patients with acute spontaneous ICH, and this increase in blood MMP-9 level is closely associated with subsequent expansion of the hematoma, perihematomal edema, and deterioration of neurologic function [8, 9]. In both rodent models and human subjects, early increase in MMP-9 expression has been confirmed in brain tissue after ICH [10, 11]. Conversely, the inhibition of MMP-9 activity was shown to attenuate ICH-induced brain injury and neurologic deficits in mice [12, 13]. Furthermore, knockout mice that lack the MMP-9 gene showed less brain edema than their wild-type littermates in a model of ICH [11]. Thus, strategies aimed at preventing the upregulation of MMP-9 expression could be therapeutic targets for secondary injury after ICH.

NF-κB pathway is known to play an essential role in inflammatory processes. The NF-κB expression has been confirmed to be upregulated early upon the onset of ICH and has been suggested to contribute to brain impairments of the tissues around the hematoma [14, 15]. As an important transcriptional regulatory factor, NF-κB participates in transcription regulation of multiple target genes, including TNF-α. Activated NF-κB promotes the transcription of TNF-α mRNA, and TNF-α in turn promotes the activation of NF-κB, resulting in a continued and enlarged inflammatory response [16]. In addition to TNF-α, the MMP-9 expression is also regulated by NF-κB. There is evidence that the NF-κB response element is located in the promoter region of the MMP-9 gene [17].

Baicalin (7-glucuronic acid, 5, 6-dihydroxyflavone) is a major flavonoid compound purified from the dry roots of *Scutellaria baicalensis* Georgi. It has been demonstrated that baicalin possesses multiple pharmacological activities including anti-oxidation, anti-tumor, and anti-inflammation [18–20]. In animal models, baicalin has been shown to attenuate ischemic brain injury possibly through anti-inflammatory and anti-apoptotic mechanisms [21, 22]. In our previous study, we demonstrated that complex prescriptions containing *S. baicalensis* Georgi were able to reduce brain edema in rat brain after ICH [23]. In the present study, we evaluated the effects of baicalin on ICH-induced brain injury in an ICH rat mode and explored the possible underlying mechanisms.

## MATERIALS AND METHODS

### Chemicals and Reagents

Baicalin was purchased from Sichuan Xieli Pharmaceutical Co. Ltd. (Chinese Drug Approval Number: H20053191, purity was 98.1 %, Chengdu, China). Collagenase VII was purchased from Sigma–Aldrich (St. Louis, MO, USA). Ten percent chloral hydrate was purchased from Qilu Pharmaceutical Co. Ltd. (Jinan, China). MMP-9 and NF-κB mouse monoclonal antibody was purchased from Santa Cruz Biotechnology (Santa Cruz, CA, USA). Phospho-IκBα (Ser32/36) and IκBα antibodies were purchased from Cell Signaling Technology (Shanghai, China). Rabbit polyclonal antibody against laminin was purchased from Lab Vision Corporation (USA). Hybond-P PVDF membrane blot was purchased from GE Healthcare Life Sciences (Beijing, China). TRIzol Reagent was purchased from Invitrogen (CA, USA).

### Animals and Experimental Proceeding

The animal experiments were conducted in accordance with the Principles of Laboratory Animal Care (NIH Publication No. 86-23, revised 1985) and approved by the Ethics Committee of The Second Hospital of Shandong University, Jinan, China.

Male Wistar rats, aged 12 weeks and weighing 320–350 g, were provided by the Laboratory Animal Center of Shandong University (Jinan, Shandong, China). Prior to the experiments, the animals were allowed to acclimate to the new environment for 1 week. During the adaptation and the experimental periods, the animals were housed in separate cages under diurnal lightning conditions with free access to food and water. ICH was induced by intracerebral administration of 0.5 U collagenase VII in 1 μL saline into the caudate nucleus of the rats at a rate of 0.2 μL/min as previously described [24]. In brief, under anesthesia with 10 % chloral hydrate (0.36 mg/g body weight, intraperitoneal), the rats were injected unilaterally into the caudate putamen with collagenase VII (0.5 U in 1 μL saline), at the following stereotactic coordinates: 0.2 mm posterior to bregma, 3.0 mm lateral to the midline, and 6.0 mm in depth below the skull. Collagenase was delivered for 5 min, and the needle was left in place for an additional 10 min to prevent any reflux. Rectal temperature was maintained at 37 ± 0.5 °C throughout the experimental and recovery periods. The sham operation rats were administered with an equal volume of saline without collagenase VII. ICH was confirmed by the appearance of hematoma in the caudate nucleus. The rats with ICH were randomly divided into four groups: group B (vehicle-treated), group C (baicalin, 25 mg/kg), group D (baicalin, 50 mg/kg), and group E (baicalin, 100 mg/kg). The rats with sham operation were used as controls (group A). Two hours after the ICH induction, the animals were injected either with saline (groups A and B) or baicalin (groups C, D, and E) through intraperitoneal injection, and the same treatments were conducted once a day thereafter. On day 1, day 3, and day 5, six rats from each group were sacrificed by decapitation under anesthesia with 10 % chloral hydrate. The brain tissues were collected from the perihematoma areas and stored at −70 °C before the assays.

### Neurobehavioral Function Evaluation

Neurobehavioral function was evaluated by a score system as described previously [25]. The score system consists of three individual tests, each with a score range of 0–4 (0 = best, 4 = worst). The maximum total score value is 12. The tests included: (1) spontaneous ipsilateral circling behavior, (2) contra-lateral forelimb and hindlimb retraction capability, and (3) ability to walk a 70-cm-long 32.4-cm-wide wood beam. The evaluation was conducted by a masked observer at day 1, day 3, and day 5 after the induction of ICH.

### Measurement of Brain Water Content

After the rats were sacrificed, the brain tissues were immediately removed. Coronal slices of 4 mm each from the frontal poles of the right brain were made. Wet weight (WW) of each slice was determined by an electronic analytic balance. Subsequently, the slices were dried in an oven at 110 °C for 24 h and weighed again to get the data of dry weights (DW). The water content (%) of the brain tissues was calculated as [(WW − DW)/WW] × 100*%*.

### Real Time RT-PCR Analysis of MMP-9 mRNA

RNA concentration was determined by UV absorbance at 260 nm. First-strand cDNA synthesis was performed with 2 μg of total RNA using random hexamers as primers in a final volume of 20 μL (5 μg/μL random hexamers, 1 mm dNTPs, 2 U/L RNasin, and 10 U/L Moloney murine leukemia virus reverse transcriptase). The reaction was carried out at 37 °C for 60-min cDNA encoding β-actin; MMP-9 was amplified from 3 to 5 μL of the cDNA reaction mixture using specific gene primers. Oligonucleotide primers for β-actin and MMP-9 were as follows: β-actin, 5′-TGACGGGGTCACCCACACTGTGCCCATC-TA-3′ (forward primer), 5′-CTAGAAGCATTTGCGGTGGACGATG-3′ (reverse primer); Mmp-9, 5′-AGTTTGGTGTCGCGGAGCAC-3′ (forward primer) and 5′-TACATGAGCGCTTCCGGCAC-3′ (reverse primer).

The amplification conditions were as follows: for MMP-9, 30 cycles of denaturation at 94 °C for 1 min, primer annealing at 55 °C for 1 min, extension at 72 °C for 2 min, and then 1 cycle of final extension at 72 °C for 5 min; for β-actin, 25 cycles of denaturation at 94 °C for 45 s, primer annealing at 60 °C for 45 s, extension at 72 °C for 2 min, and then 1 cycle of final extension at 72 °C for 5 min. The expression of β-actin was be used as an internal control for the assay of a constitutively expressed gene.

### Western Blot Analysis

Western blotting was performed as previously described [26]. Briefly, the brain tissues were homogenized in radio immunoprecipitation assay (RIPA) lysis buffer containing protease and phosphatase inhibitors. The proteins were separated by electrophoresis on a 10 % SDS–PAGE gel and transferred to a Hybond-P PVDF membrane. Subsequently, the membrane was blocked in 5 % nonfat milk and incubated with appropriate primary antibodies (RelA/p65, 1:1,000; MMP-9, 1:1,000) overnight at 4 °C, followed by incubation with the peroxidase-conjugated rabbit anti-goat secondary antibody (1:1,000 dilution) for 1 h at room temperature. After washing with PBS, the bound primary antibody was visualized with the Enhanced Chemiluminescence System from Amersham (Piscataway, NJ, USA) and exposed to film. The same membrane was probed for β-actin for loading control. The relative density of RelA/p65 to β-actin was analyzed with the TotalLab TL120 Image software (TotalLab Life Science Analysis Essentials, Greensboro, NC, USA).

### Enzyme-Linked Immunosorbent Assay for IL-1β and IL-6

The brain tissues were collected from the perihematoma areas. The samples were weighed. After homogenization, the lysate was mixed with equal volume of RIPA buffer and put on ice for 30 min. After centrifugation (12,000 rotations/min for 20 min), the supernatant was collected for IL-1β and IL-6 measurements using enzyme-linked immunosorbent assay (ELISA) kits (Wuhan Boster Biological Technology, Wuhan, China) according to the manufacturer’s instructions. The results were expressed as picograms per milligram of tissues.

### Determination of Blood–Brain Barrier Permeability

Evans blue dye (100 mg/kg, Sigma) was injected into the femoral vein. After 1 h, the rats were perfused with saline through the left ventricle until the perfusion fluid obtained from the right atrium became colorless. After decapitation under anesthesia with ether, the brain tissues were collected from the perihematoma areas. The samples were weighed and soaked in 50 % trichloroacetic acid solution. After homogenization and centrifugation (12,000 rotations/min for 20 min), the supernatant was diluted with ethanol (1:3). The fluorescence intensity was measured at 620 and 680 nm for excitation and emission. The concentration of the Evans blue dye in the brain tissue was calculated according to a linear standard curve obtained from known amounts of the dye. The tissue content of the Evans blue dye was expressed in micrograms per gram of tissues.

### Statistical Analysis

All data were presented as means ± SD and analyzed with one-way analysis of variance and Student’s *t* test. *P* value <0.05 was considered statistically significant.

## RESULTS

### Effect of Baicalin on Neurobehavioral Functions

**Fig. 1:**
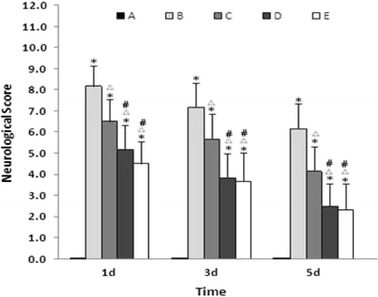
Baicalin improved neurobehavioral deficits following ICH. The aggregate score represents the average neurobehavioral function per animal (*n* = 6 for each group). *Asterisk* denotes *P* < 0.01 vs. group A; *triangle*, *P* < 0.05 vs. group B; and *number sign*, *P* < 0.05 vs. group C. Group A is the control group; group B, vehicle-treated ICH; group C, baicalin (25 mg/kg)-treated ICH; group D, baicalin (50 mg/kg)-treated ICH; and group E, baicalin (100 mg/kg)-treated ICH.

In the ICH model group, around 10 % of the rats would die in 24 h following the operation. In the sham operation group, all rats survived. Neurobehavioral deficits were evaluated using a scoring system. After the induction of ICH, all ICH groups showed neurobehavioral deficits as compared with the sham operation group. The neurobehavioral deficits attenuated over time in each group, suggestive of self-recovery ability of the rats. At each time point, baicalin significantly improved the neurobehavioral function in a dose-dependent manner (Fig. 1).

### Effect of Baicalin on Brain Edema after ICH

**Fig. 2:**
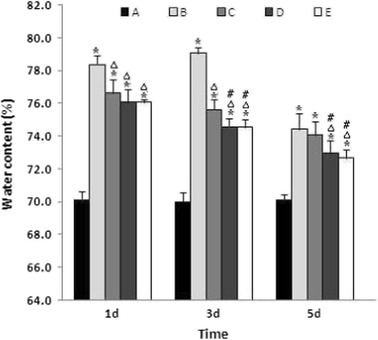
Baicalin reduced brain water content following ICH. Brain water content was measured as described in “MATERIALS AND METHODS”. The quantitation represents the average value of the brain water content per animal (*n* = 6 for each group). *Asterisk* denotes *P* < 0.01 vs. group A; *triangle*, *P* < 0.01 vs. group B; and *number sign*, *P* < 0.05 vs. group C. Group A is the control; group B, vehicle-treated ICH; group C, baicalin (25 mg/kg)-treated ICH; group D, baicalin (50 mg/kg)-treated ICH; group E, baicalin (100 mg/kg)-treated ICH.

Brain edema was evaluated by measuring the brain water content (Fig. 2). After the induction of ICH, the brain water content in the vehicle-treated ICH group (group B) increased significantly on day 1 compared to the sham operation group (group A) and peaked on day 3. At each time point, the brain water content in group B was significantly higher than that in the control group (*P* < 0.01, respectively). On day 3 and day 5 after ICH, baicalin significantly reduced the brain water content in a dose-dependent manner. On day 10, the brain water contents in all the ICH groups were still higher than that of the control group, whereas the brain water content of the vehicle-treated ICH group was not significantly different from those of the baicalin-treated groups.

### Effect of Baicalin on MMP-9 Protein and mRNA Expression

**Fig. 3:**
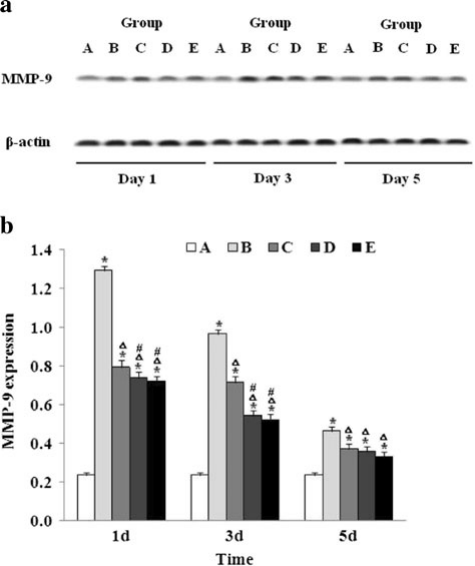
Baicalin suppressed MMP-9 protein expression following ICH. **a** Representative experiments of western blot for the MMP-9 expression (*n* = 6 for each group). **b** At each time point, the MMP-9 expression was determined by western blot as described in “MATERIALS AND METHODS”. The quantitation represents the average relative ratio of MMP-9 protein to β-actin per animal (*n* = 6 for each group). *Asterisk* signifies *P* < 0.01 vs. group A; *triangle*, *P* < 0.01 vs. group B; and *number sign*, *P* < 0.05 vs. group C. Group A is the control; group B, vehicle-treated ICH; group C, baicalin (25 mg/kg)-treated ICH; group D, baicalin (50 mg/kg)-treated ICH; group E, baicalin (100 mg/kg)-treated ICH.

MMP-9 is known to play an essential role in the maintenance of the integrity of blood–brain barrier and has been implicated in brain edema formation. In the present study, we first determined the MMP-9 protein expression in the brain tissues of the rats after ICH by western blotting. As shown in Fig. 3, the MMP-9 expression was significantly increased on day 1 in the vehicle-treated ICH mice (group B), compared to that in the sham operation group (group A), and reached a peak on day 3 after ICH. Baicalin attenuated the MMP-9 expression in a dose-dependent manner at each time point.

**Fig. 4:**
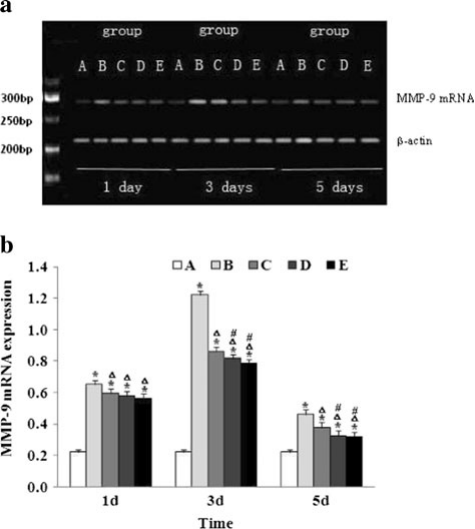
Baicalin suppressed MMP-9 mRNA expression following ICH. **a** Representative experiments of RT-PCR for MMP-9 mRNA expression (*n* = 6 for each group). **b** At each time point, the MMP-9 mRNA expression was determined by RT-PCR as described in “MATERIALS AND METHODS”. The quantitation represents the average relative ratio of MMP-9 mRNA to β-actin per animal (*n* = 6 for each group). *Asterisk* signifies *P* < 0.01 vs. group A; *triangle*, *P* < 0.01 vs. group B; and *number sign*, *P* < 0.05 vs. group C. Group A is the control; group B, vehicle-treated ICH; group C, baicalin (25 mg/kg)-treated ICH; group D, baicalin (50 mg/kg)-treated ICH; group E, baicalin (100 mg/kg)-treated ICH.

The MMP-9 mRNA expression was analyzed by RT-PCR. As shown in Fig. 4, the MMP-9 mRNA level was significantly increased on day 1 in the vehicle-treated ICH mice (group B) compared to that in the sham operation group (group A) and reached a peak on day 3. Baicalin inhibited ICH-induced MMP-9 mRNA expression in a dose-dependent manner.

### Effect of Baicalin on NF-κB Expression

**Fig. 5:**
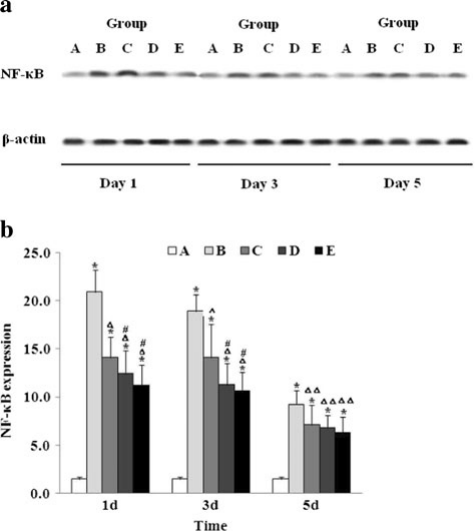
Baicalin suppressed NF-κB protein expression following ICH. **a** At each time point, the NF-κB expression was determined by western blot as described in “MATERIALS AND METHODS”. The quantitation represents the average relative ratio of NF-κB protein to β-actin per animal (*n* = 6 for each group). *Asterisk* indicates *P* < 0.01 vs. group A; *triangle*, *P* < 0.05 vs. group B; double triangle, *P* < 0.01 vs. group B; and *number sign*, *P* < 0.05 vs. group C. Group A is the control group; group B, vehicle-treated ICH; group C, baicalin (25 mg/kg)-treated ICH; group D, baicalin (50 mg/kg)-treated ICH; group E, baicalin (100 mg/kg)-treated ICH. **b** Representative experiments of western blot for the NF-κB expression (*n* = 6 for each group).

The effects of baicalin on the NF-κB (RelA/p65) expression and activity were determined by western blotting. As shown in Fig. 5, the RelA/p65 expression was significantly increased on day 1 in the vehicle-treated ICH mice (group B) compared to that in the sham operation group (group A), in which the level decreased over time. Baicalin inhibited the NF-κB expression in a dose-dependent manner at each time point.

### Effect of Baicalin on IL-1β and IL-6 Production

**Fig. 6:**
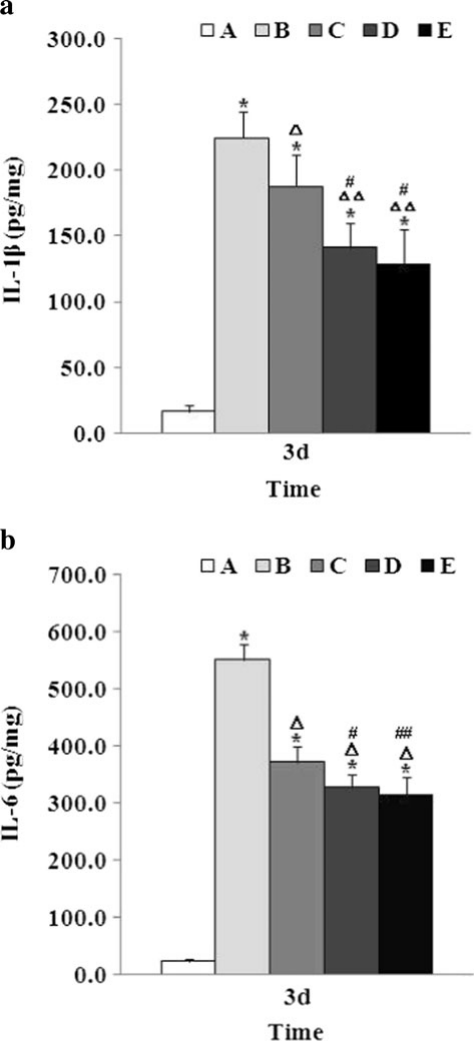
Baicalin reduced IL-1β and IL-6 production following ICH. IL-1β and IL-6 levels were determined by enzyme-linked immunosorbent assay. Group A is the control group; group B, vehicle-treated ICH; group C, 25 mg/kg baicalin-treated ICH; group D, 50 mg/kg baicalin-treated ICH; group E, 100 mg/kg baicalin-treated ICH. **a** *Asterisk* indicates *P* < 0.01 vs. group A; *triangle*, *P* < 0.05 vs. group B; *double triangle*, *P* < 0.01 vs. group B; and *number sign*, *P* < 0.01 vs. group C. **b** *Asterisk* indicates *P* < 0.01 vs. group A; *triangle*, *P* < 0.01 vs. group B; *number sign*, *P* < 0.05 vs. group C; and *double number sign*, *P* < 0.01 vs. group C.

The activation of the NF-κB signaling pathway is known to play an essential role in inflammatory processes. In the present study, additional experiments were conducted to observe the effects of baicalin on the production of pro-inflammatory cytokines including IL-1β and IL-6 in the brain tissues at 72 h following ICH. The IL-1β and IL-6 levels were determined by ELISA. As shown in Fig. 6a, b, following ICH, the production of IL-1β and IL-6 was significantly increased in the brain tissues around the perihematoma areas. Baicalin reduced the IL-1β and IL-6 production in a dose-dependent manner.

### Effect of Baicalin on Blood–Brain Barrier Permeability

**Fig. 7:**
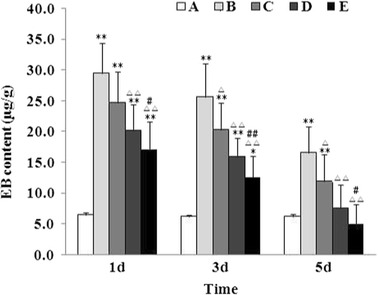
Baicalin attenuated blood–brain barrier permeability following ICH. Blood–brain barrier permeability was determined by Evans blue leakage method. Group A is the control; group B, vehicle-treated ICH; group C, 25 mg/kg baicalin-treated ICH; group D, 50 mg/kg baicalin-treated ICH; group E, 100 mg/kg baicalin-treated ICH. *Asterisk* denotes *P* < 0.05 vs. group A; *double asterisk*, *P* < 0.01 vs. group A; *triangle*, *P* < 0.05 vs. group B; *double triangle*, *P* < 0.01 vs. group B; *number sign*, *P* < 0.05 vs. group C; and *double number sign*, *P* < 0.01 vs. group C.

As MMP-9 is a key regulator of the BBB permeability, next we determined the effect of baicalin on the blood–brain barrier permeability by Evans blue leakage method. As shown in Fig. 7, the BBB permeability significantly increased and peaked at 24 h after ICH, which was attenuated by baicalin dose dependently.

## DISCUSSION

In the present study, we clearly demonstrated that baicalin significantly attenuated brain edema after ICH in a collagenase-induced ICH rat model. Additionally, our data showed that the beneficial effect of baicalin is associated with the inhibition of the NF-κB pathway and the suppression of the MMP-9 expression.

ICH is a common and devastating cerebral disease with a higher morbidity and mortality than ischemic stroke [26]. ICH-induced brain injury involves multiple mechanisms. The primary injury is due to the disruption of adjacent tissue and mass effect. The secondary brain damage occurs as a result of hematoma, inflammation, local release of ROS, and perihematomal edema [27]. Edema secondary to ICH has been suggested to be a major reason for neurological deterioration after the first 24 to 48 h from the onset of symptoms [28]. Drugs capable of reducing brain edema demonstrated improvements in neurological functions in both animal models and human subjects after ICH [25, 29].

The results from the present study showed that collagenase challenge robustly increased brain water content in perihematoma regions within 24 h after ICH and reached a peak on day 3. These results are in consistent with previous observations [30]. In addition, we demonstrated that baicalin significantly reduced ICH-induced brain edema in a dose-dependent manner. In a normal brain, the BBB prevents the flow of water into the brain due to hydrostatic pressure gradients. After ICH, breakdown of BBB plays an important role in edema formation [31]. MMPs are a family of proteolytic enzymes that are closely associated with the integrity of the blood–brain barrier. MMPs, in particular MMP-9, have been implicated in ICH-induced secondary brain injury and edema formation in both experimental models and humans [32, 33]. To explore the possible mechanism by which baicalin reduces ICH-induced brain edema, we determined the effect of baicalin on the MMP-9 expression. Our results showed that the MMP-9 expression was significantly increased within 24 h after ICH and peaked on day 3. This upregulation of the MMP-9 expression could be due to a transcriptional mechanism as the mRNA level was increased. Along with the increase in the MMP-9 expression, the blood–brain barrier permeability was increased after ICH, as evaluated by Evans blue leakage method. This was consistent with the hypothesis that MMP-9 may promote edema formation through increasing the permeability of the blood–brain barrier after ICH. Baicalin treatment not only inhibited ICH-induced MMP-9 expression at both protein and mRNA levels but also significantly reduced the blood–brain barrier permeability in a dose-dependent manner.

NF-κB is a ubiquitous transcription factor and a member of a family of proteins that are critical regulators of inflammation. In unstimulated cells, NF-κB exists in the cytoplasm as a dimer predominantly composed of the p50 and p65 (RelA) subunits and inactivated by IκB. In response to external pathogenic stimuli, including cytokines, reactive oxygen species, and specific kinases, IκB is phosphorylated, which leads to its degradation and dissociation from NF-κB. The free NF-κB then migrates into the cell nucleus, where it binds to the specific NF-κB response elements in the promoters of target genes [34]. Previous studies have shown that NF-κB is rapidly activated in perihematomal brain after ICH and plays an important role in ICH-induced brain damages [34, 35]. In line with the previous studies, our results showed that ICH significantly upregulated the NF-κB (RelA) expression, which peaked on 24 h after ICH. Typically, the activation of NF-κB results in the transcriptional induction of genes for many pro-inflammatory substances, such as cytokines, chemokines, adhesion molecules, and inflammatory enzymes [15]. In the present study, we determined the production of IL-1β and IL-6 in the tissues around the perihematoma areas. Our results showed the levels of these pro-inflammatory cytokines were increased.

Recent studies have shown that NF-κB also plays an important role in the regulation of MMP-9 expression [17, 36]. Therefore, NF-κB-induced release of pro-inflammatory substances and upregulation of MMP-9 may play important roles in brain injury after ICH.

Baicalin is a major compound isolated from the dry roots of *S. baicalensis*, which is a widely used traditional Chinese herb with proven anti-inflammatory property [37]. To test the hypothesis that baicalin may protect against brain injury through the regulation of inflammatory process, we studied its effect on NF-κB expression after ICH. Our results showed that baicalin inhibited ICH-induced NF-κB expression in a dose-dependent manner. In addition, baicalin also reduced the production of IL-1β and IL-6 in the tissues around the perihematoma areas.

Taken together, our results demonstrated that baicalin effectively reduces perihematomal edema after ICH in rats, raising the possibility of the use of baicalin as a potential drug for the treatment of ICH. The mechanisms underlying the effect of baicalin may be partly through the inhibition of the NF-κB pathway and the suppression of the MMP-9 expression.
